# A Dual-Wavelength Phosphorescent Anti-Counterfeiting Copolymer Containing Eu(III) and Tb(III)

**DOI:** 10.3390/polym15030736

**Published:** 2023-01-31

**Authors:** Hui Zhao, Zihao Wang, Yongchao Wang, Jiandong Guo, Aiqin Zhang, Husheng Jia, Bingshe Xu

**Affiliations:** 1 Key Laboratory of Interface Science and Engineering in Advanced Materials, Taiyuan University of Technology, Ministry of Education, Taiyuan 030024, China; 2 Shanxi Research Institute of Huairou Laboratory, Taiyuan 030001, China; 3 College of Textile and Engineering, Taiyuan University of Technology, Taiyuan 030006, China; 4 Shanxi-Zheda Institute of Advanced Materials and Chemical Engineering, Taiyuan 030000, China

**Keywords:** rare earth, bonded copolymer, luminescent properties, dual-wavelength, phosphorescent anti-counterfeiting

## Abstract

The anti-counterfeiting technology of banknotes, bills and negotiable securities is constantly copied, and it is urgent to upgrade its anti-counterfeiting technology. In view of the defect of easy replication of single-wavelength anti-counterfeiting technology, the bonded copolymer PMEuTb was synthesized, employing the technique of first coordination and then polymerization. The molecular structure of copolymer PMEuTb was confirmed by infrared spectrum and UV-vis absorption spectrum. The internal mechanism of negative correlation between initiator concentration and number-average molecular weight *M*_n_ of the copolymer was revealed, and the positive correlation between *M*_n_ and luminescent behavior of the copolymer was analyzed. The luminescent properties of copolymer PMEuTb with initiator amount of 0.1% were investigated, the copolymer PMEuTb exhibits dual-wavelength emission of green light and red light under the excitation of ultraviolet light at 254 nm and 365 nm. The copolymer has the lifetime of 1.083 ms at ^5^D_4_–^7^F_5_ transition and 0.665 ms at ^5^D_0_–^7^F_2_ transition, which belongs to phosphorescent emitting materials. The copolymer remains stable at 240 °C, and variable temperature photoluminescent spectra demonstrate the luminescent intensity remains 85% at 333 K, meeting the requirements of room temperature phosphorescent anti-counterfeiting materials. The luminescent patterns made by standard screen printing display the green and cuticolor logo at 254 nm and 365 nm, respectively, indicating that the bonded phosphors PMEuTb has potential application in phosphorescent anti-counterfeiting.

## 1. Introduction

With the development of the modern science and technology industry, fake products flood into the market, especially the counterfeiting of banknotes, bills and securities, which seriously disrupt the market order. At the same time, because the anti-counterfeiting technology is constantly copied, fake products are spreading as a sort of “economic cancer” [1,2,3,4]. As a simple anti-counterfeiting technology, anti-counterfeiting ink has become the spotlight in money, securities, anti-counterfeiting documents and packaging anti-counterfeiting materials, owing to good invisibility, being easy to identify, and wavelength and color control [5,6,7,8,9]. Some rare earth ions show pure luminescent characteristics of fixed wavelength and narrow emission, high color purity and good color saturation due to the 4*f* electron being shielded by the 5*s*^2^5*p*^6^ electron, becoming the first choice of luminescent center of anti-counterfeiting materials [10,11,12]. The bonded rare earth polymer phosphors have both excellent luminescent performance of a rare earth complex and good film forming performance of a polymer [13,14,15,16,17,18], which has become a research focus of anti-counterfeiting ink.

Jia et al. [19] synthesized an amphiphilic multiblock aromatic copolymer by, first, polymerization and then coordination, which was used as a ligand to sensitize Tb^3+^ and Eu^3+^ to obtain a monochromatic anti-counterfeiting macromolecular complex. Zhao et al. [20] developed three kinds of Eu, Tb and Gd coordination polymers used in anti-counterfeiting, employing the solvothermal synthesis method. Xie et al. [21] synthesized the copolymer of N-(2-aminoethyl)-3-aminopropyl trimethoxysilane (AEAPS) and citric acid by solution polymerization and then coordinated with Eu^3+^ to obtain AEAPS-Eu (III) anti-counterfeiting materials that can emit specific colors (blue and red). Jung et al. [22] developed the macromolecule ligand 2,6-pyridinediamine-bispropylheptylisobutyl POSS (PDC-POSS), and then coordinated with Eu^3+^ to synthesize PDC-POSS: Eu^3+^ macromolecular complex, which can be used for red light anti-counterfeiting for ultraviolet light identification.

Because the macromolecular complex prepared by first polymerization and then coordination has a fixed excitation wavelength, generally, only anti-counterfeiting materials with a fixed wavelength were obtained. The anti-counterfeiting materials are easy to be copied, and their security is difficult to meet the anti-counterfeiting requirements. The first coordination and then polymerization can first coordinate ligands with different excitation wavelengths to different rare earth ions, and then copolymerize different rare earth complex monomers to obtain bonded single-wavelength [23,24], dual-wavelength and multi-wavelength anti-counterfeiting materials. So far, there are few reports on dual-wavelength or multi-wavelength bonded anti-counterfeiting materials employing the technique of first coordination and then polymerization.

In this work, dual-wavelength excitation bonded polymer phosphors have been copolymerized with the Eu^3+^ and Tb^3+^ complex monomers and methyl methacrylate, employing the technical route of first coordination and then polymerization. The structure and thermal properties of the copolymer were characterized by infrared spectrum, UV-vis absorption spectrum and thermogravimetry. The influence of relative molecular weight on the luminescent properties of the copolymer was analyzed and revealed. The luminescent behavior of the phosphors with temperature changes was studied. Finally, the phosphors were applied to the field of counterfeiting and showed excellent phosphorescent anti-counterfeiting performance.

## 2. Experiments

### 2.1. Experimental Materials

Methyl ethacrylate (MMA), 2,2-azoisobutyronitrile (AIBN), 2-thenoyltrifluor oacetone(TTA), 1,10-phenanthroline (phen), 4-benzoylbenzoic acid (*p*-BBA) and undecylenic acid (UAH) were obtained from Alfa Aesar. EuCl_3_ and TbCl_3_ were purchased from Shanghai Diyang Co. Ltd. NaOH was purchased from Tianjin Dengfeng Chemical Reagent Factory. Dimethyl sulfoxide (DMSO) and N,N-dimethylformamide (DMF) were obtained from Tianjin Ruijinte Chemicals Co. Ltd. Anhydrous methanol and anhydrous ethanol were purchased from Tianjin Beichen Founder Reagent Factory.

### 2.2. Preparation of Eu(TTA)_2_(phen)UA and Tb(p-BBA)_3_UA Complex Monomers

**Figure 1:**
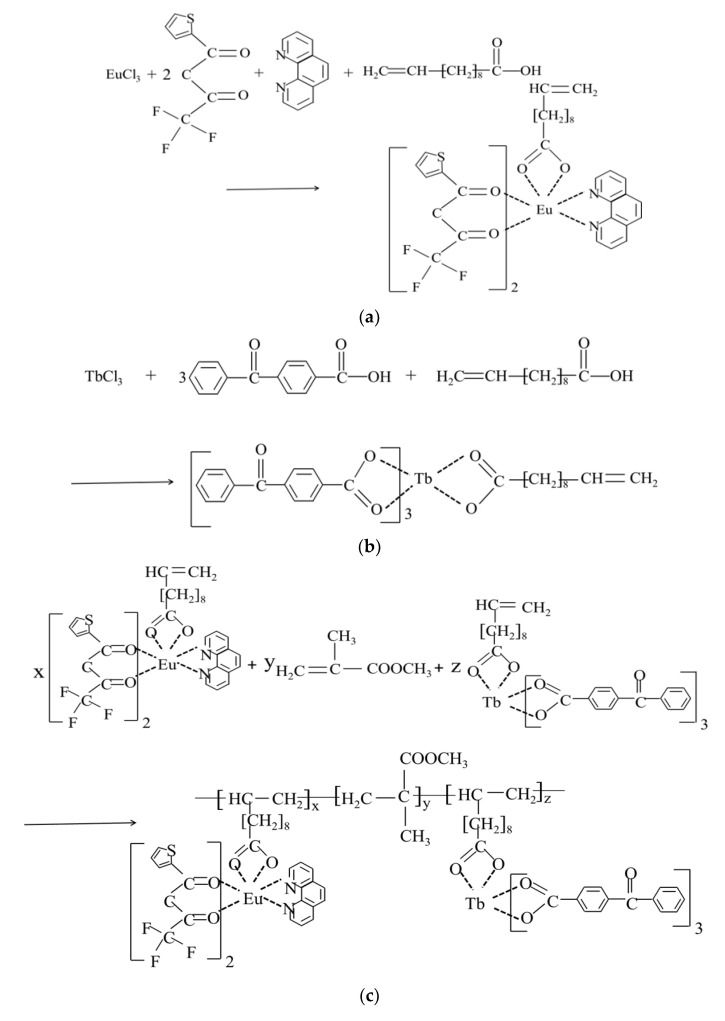
Synthetic route of the complex monomers Eu(TTA)_2_(phen)UA (**a**), Tb(*p*-BBA)_3_UA (**b**) and the copolymer PMEuTb (**c**).

The preparation of the complex monomer Eu(TTA)_2_(phen)UA was similar to that of Tb(*p*-BBA)_3_UA. Take the complex Eu(TTA)_2_(phen)UA as an example, 1 mmol of EuCl_3_, 2 mmol of TTA, 1 mmol of phen and 1 mmol UAH were dissolved in 10 mL of absolute ethanol, respectively. Next, the TTA and phen ethanol solution was added to a three-neck flask and heated at 50 °C. Then, the EuCl_3_ ethanol solution was added drop by drop, and the pH of the solution was adjusted to 4~5 using 1 mol/L NaOH ethanol solution. After half an hour of reaction, the UAH solution was added dropwise, and pH was adjusted to 6.5~7. At this time, a large amount of white precipitates were generated. The reaction was kept for 4 h. When the reaction was complete, the reaction product was filtered, washed with anhydrous ethanol 5~6 times and then dried at 80 °C for 5 h to obtain a white solid powder. The product was Eu(TTA)_2_(phen)UA. The synthetic routes of the proposed complex monomer Eu(TTA)_2_(phen)UA and Tb(*p*-BBA)_3_UA are shown in Figure 1a,b. Elemental analysis for Eu(TTA)_2_(phen)UA is as follows: element analytical (calc.) C 47.92% (48.19%), H 3.93% (4.01%), N 2.90% (2.88%), S 6.58% (6.59%). Elemental analysis for Tb(*p*-BBA)_3_UA is as follows: element analytical (calc.) C 62.17% (62.54%), H 4.98% (4.52%).

### 2.3. Preparation of the Copolymer PMEuTb

The appropriate amount of the complex monomer Eu(TTA)_2_(phen)UA and Tb(*p*-BBA)_3_UA, methyl methacrylate (MMA) were dissolved in 3 mL DMSO and dispersed by ultrasound for 20 min until being dissolved completely. The solution was added into a test tube with a branch and was bubbled to remove oxygen with nitrogen for 30 min. Then, AIBN was added to the test tube by 0.1%, 0.2%, 0.3%, 0.4% and 0.5% of the total molar mass of the monomers, respectively. The solution was heated at 75 °C in a water bath, and nitrogen was continued for 2 h. When the system was viscous, nitrogen flow was stopped, the system was sealed and the reaction continued at 75 °C for 48 h. The product obtained from the reaction was poured into 100 mL of anhydrous methanol, resulting in a white flocculent precipitate. After alternately washing 6 times with DMSO and methanol to remove unreacted monomers and impurities in the product, the bonded copolymer PMEuTb with a different relative molecular weight was obtained by drying at 75 °C for 12 h. The synthetic route of proposed copolymer PMEuTb is presented in Figure 1c.

### 2.4. Measurements

Elemental analysis of the complexes was conducted on a PerkinElmer Elemental Analyzer. The infrared spectrum was measured in the range of 4000~400 cm^−1^ using the KBr disk method by a Nicolet 7199B Fourier Transform Infrared Spectrometer. Using a Cary-300 VARIAN, the UV-vis absorption spectrum of the complex was measured by 1 × 10^−4^ mol/L DMSO solution. The thermogravimetric curves were measured by using a NETZSCH TG209F3 thermogravimetric analyzer made in Germany, with nitrogen as the protective gas and a heating rate of 10 °C/min. The molecular weight and molecular weight distribution were measured by a Waters 410 gel permeation chromatograph with tetrahydrofuran as the eluent. The photoluminescent spectrum was measured in solid state, employing a Hitachi F-4700 fluorescence spectrophotometer. The photoluminescent spectrum of variable temperature was detected in the range of 20~180 °C by a PerkinElmer LS55 spectrofluorophotometer. Using the time-correlated single photon counting (TCSPC) method, the luminescent lifetime was determined by an Edinburgh Instruments FLS980 steady-state/Transient fluorescence spectrometer.

## 3. Results and Discussion

### 3.1. IR Spectra of the Copolymer PMEuTb

**Figure 2:**
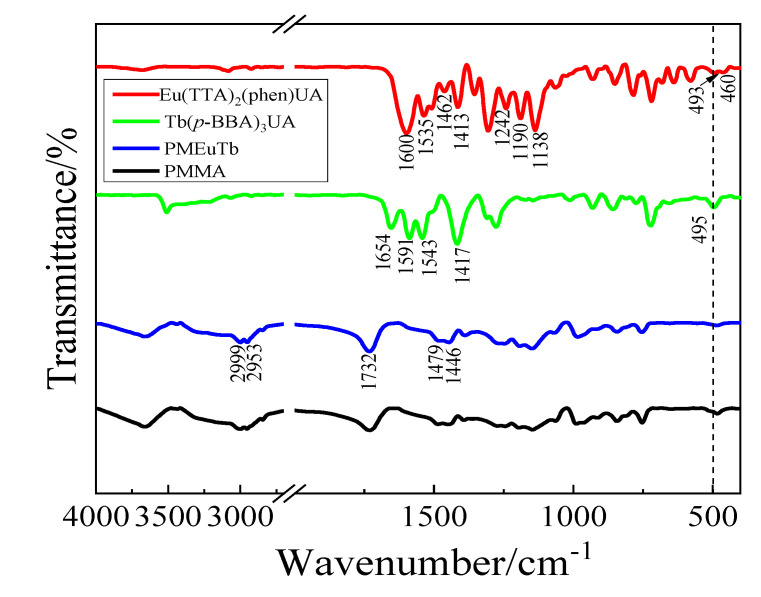
IR spectra of the complexes Eu(TTA)_2_(phen)UA and Tb(*p*-BBA)_3_UA, the copolymer PMEuTb and the homopolymer PMMA.

The infrared spectra of the complexes Eu(TTA)_2_(phen)UA and Tb(p-BBA)_3_UA, and polymers PMMA and PMEuTb, were recorded by the KBr tablet method, as shown in Figure 2. The infrared characteristic data are listed in Table 1. In the complex Eu(TTA)_2_(phen)UA, 1242, 1190 and 1138 cm^−1^ belong to the antisymmetric and symmetric stretching vibration multiplets of CF_3_ in the ligand TTA, the peak at 1588 cm^−1^ is attributed to the stretching vibration of C=O in TTA redshifts to 1600 cm^−1^ in the complex and the characteristic peak at 493 cm^−1^ is assigned to the stretching vibration of Eu-O, indicating that the C=O in TTA has coordinated with the Eu(III) ions. The peak at 1462 cm^−1^ is assigned to the stretching vibration of C=N in the ligand phen, and the weak absorption peak at 460 cm^−1^ belongs to the stretching vibration of Eu-N, indicating that phen has coordinated with the Eu(III) ions. The peaks at 1535 cm^−1^ and 1413 cm^−1^ assigned to the antisymmetric and symmetric stretching vibration of carboxylate COO- are detected in the complex, owing to the loss of H^+^ ions from -COOH, indicating that UAH coordinates successfully with the Eu(III) ions.

It can be seen from the infrared spectra of the complex Tb(p-BBA)_3_UA that the peak at 1591 cm^−1^ is attributed to the stretching vibration of carbonyl C=O from the ligand p-BBA, the stretching vibration peaks of C=O at 1680 cm^−1^ and C-O at 1289 cm^−1^ in the ligand both disappear in the complex, the antisymmetric and symmetric stretching vibration of carboxylate COO- are probed at 1543 cm^−1^ and 1417 cm^−1^ and the characteristic peak at 495 cm^−1^ is assigned to the stretching vibration of Tb-O. The results indicate that the ligand p-BBA has coordinated with the Tb(III) ions. The C=C stretching vibration frequency is detected at 1654 cm^−1^, indicating that UAH has coordinated with the Tb(III) ions.

As can be seen from Figure 2, homopolymer PMMA has a similar infrared spectrum to copolymer PMEuTb. In the copolymer PMEuTb, the antisymmetric and symmetric stretching vibration peaks of CH_3_ are located at 2999 cm^−1^ and 2953 cm^−1^, the asymmetric and symmetric variable angle vibration peaks are located at 1479 cm^−1^ and 1446 cm^−1^ and the characteristic absorption peak at 1732 cm^−1^ is assigned to the stretching vibration peak of ester carbonyl C=O. It should be noted that the characteristic peaks of the complexes were not detected in the copolymer PMEuTb, in that the content of the complexes was low (<5%).

### 3.2. UV-vis Absorption Spectra of the Copolymer PMEuTb

**Figure 3:**
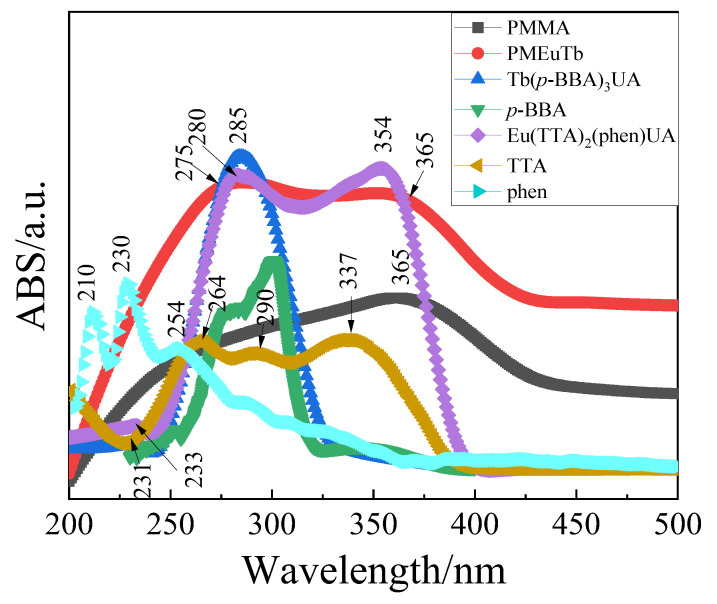
The UV-vis absorption spectra of the complex Eu(TTA)_2_(phen)UA,Tb(*p*-BBA)_3_UA, homopolymer PMMA and copolymer PMEuTb.

The UV-vis absorption spectra of Eu(TTA)_2_(phen)UA, Tb(*p*-BBA)_3_UA, TTA, phen, *p*-BBA, PMMA and PMEuTb were measured using dimethyl sulfoxide as a solvent at a concentration of 1 × 10^−4^ mol/L, as shown in Figure 3. It can be seen that there are mainly three absorption peaks in the complex Eu(TTA)_2_(phen)UA. The absorption peaks at 210, 230 and 254 nm in the free ligand phen are attributed to the π-π* transition and *n*-π* transition of biphenyl structure. The absorption peak at 230 nm in the ligand phen redshifts to 233 nm in the complex. The two absorption peaks at 280 nm and 354 nm in the complex belong to the absorption of the ligand TTA at 264, 290 and 337 nm. However, compared with the ligand TTA, the absorption peaks generate redshift in the complex, which is caused by the formation of a larger conjugate system after the formation of the complex. From the complex Tb(*p*-BBA)_3_UA, it is clear that the absorption peak at 240~322 nm in the ligand p-BBA redshifts to 244~330 nm in the complex Tb(*p*-BBA)_3_UA, which is caused by the formation of a larger conjugate system after the formation of the complex. The weak peak at 231 nm belongs to B absorption band of the benzene ring in the ligand *p*-BBA. Since the ligand UA is an aliphatic compound, it does not contain rigid groups such as benzene rings, and has poor light absorption ability, so it does not show the absorption of UAH in the complexes.

Compared with the homopolymer PMMA, after the introduction of the complexes Eu(TTA)_2_(phen)UA and Tb(*p*-BBA)_3_UA, the UV-vis absorption spectrum of the obtained copolymer PMEuTb shows not only the absorption of polymer matrix PMMA, but also the absorption of the two complexes. The overall peak shape of the copolymer PMEuTb is similar to that of PMMA. However, the intensity of the absorption peak at 275 nm is significantly enhanced, which is represented by the junction absorption of Tb(p-BBA)_3_UA, Eu(TTA)_2_(phen)UA and PMMA. The intensity at 365 nm is also enhanced, which is attributed to the absorption of the complex Eu(TTA)_2_(phen)UA and PMMA. These results indicate that the complexes Eu(TTA)_2_(phen)UA and Tb(p-BBA)_3_UA have copolymerized with methyl methacrylate to form copolymer PMEuTb. It was further proved that, by introducing a small amount of complex monomer, the copolymer shows strong UV absorption characteristic peaks of the complexes and the position of the characteristic peaks remain, indicating that the complex not only participates in the copolymerization, but also is difficult to dissociate after polymerization [25]. At the same time, it also proves the correctness of inference that the complex monomers are not detected in PMEuTb infrared spectra of Figure 2 in the low content of the complex monomers.

### 3.3. Thermal Stability of the Copolymer PMEuTb

**Figure 4:**
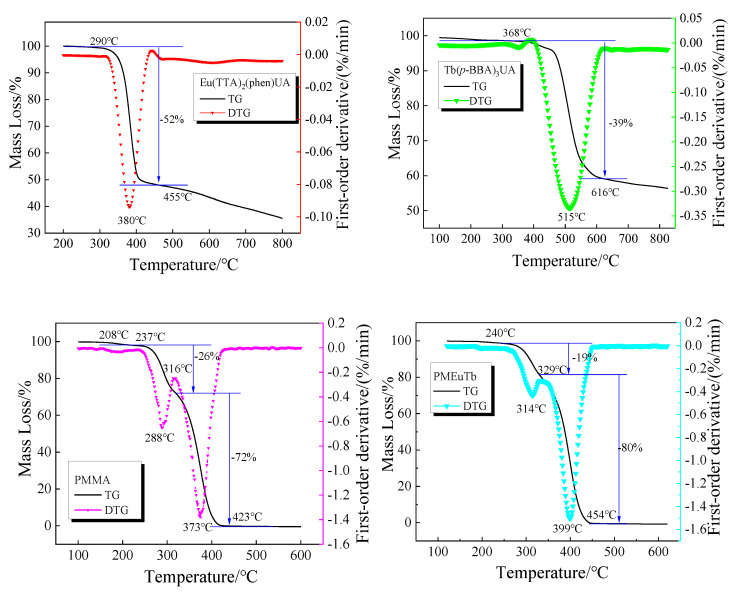
TG–DTG curves of Eu(TTA)_2_(phen)UA,Tb(*p*-BBA)_3_UA, PMMA and PMEuTb.

The TG curves of Eu(TTA)_2_(phen)UA, Tb(p-BBA)_3_UA, PMMA and PMEuTb were measured in a dynamic N_2_ atmosphere at a rate of 10 °C/min, and the DTG curves were obtained by first-order differentiation of the TG curve, as shown in Figure 4. It can be learned from the TG curves that the initial decomposition temperature of the complex Eu(TTA)_2_(phen)UA is 290 °C, and no mass loss occurs before this temperature, indicating that the complex Eu(TTA)_2_(phen)UA does not contain coordination water or water. The weight loss starts from 290 °C, the weight loss rate of the complex is 52% and the maximum weight loss rate occurs at 380 °C. At this stage, the ligands TTA and UA are mainly decomposed, being consistent with the theoretical decomposition rate of 55%. The residue is mainly stable Eu_2_O_3_.The decomposition of the complex Tb(p-BBA)_3_UA can be divided into three stages: The first weight loss stage is before 368 °C, and the weight loss rate of the complex is 1%, indicating that the complex contains trace water by confirming an OH^−^ characteristic absorption peak at 3400 cm^−1^ in the infrared spectra. At the stage of 368~616 °C, the maximum weight loss rate is located at 515 °C, and the weight loss rate at this stage is 39%. This stage is mainly the decomposition of the ligands p-BBA and UAH. After 616 °C, the complex slowly loses 4% of its mass, at which the complex decomposes completely and the remainder is Tb_2_O_3_.

The thermal property changes of the copolymer PMEuTb are similar to those of the homopolymer PMMA, and can be divided into three stages: In the first stage, the thermal properties are stable before 240 °C and its weight loss is zero due to the little impurity. The initial decomposition temperature of the copolymer PMEuTb increases from 208 °C of the homopolymer to 240 °C, indicating that, after the introduction of complex Eu(TTA)_2_(phen)UA and Tb(p-BBA)_3_UA, the rigid groups such as benzene rings are introduced into the side chain of the molecular chain of the copolymer, and the overall rigidity of the molecular chain is improved, leading to the increase of the initial decomposition temperature. The second stage occurs between 240 and 329 °C and the maximum thermal decomposition rate occurs at 314 °C. Compared with 26% weight loss of PMMA, PMEuTb has 19% weight loss, indicating that PMEuTb contains less small molecular polymer. The last stage occurs between 329 and 454 °C, with obvious weight loss, complete decomposition at 454 °C and the maximum decomposition rate at 399 °C. This stage is mainly the depolymerization of high molecular weight PMEuTb “zipper” and the decomposition of complex monomers. Compared with PMMA, the temperature at maximum decomposition rate is increased by 26 °C, indicating that the introduction of complexes is beneficial to improve the thermal stability of PMMA.

### 3.4. Luminescent Properties of the Complexes Eu(TTA)_2_(phen)UA and Tb(p-BBA)_3_UA

**Figure 5:**
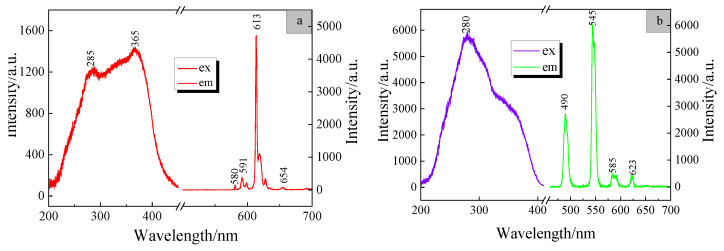
The luminescent spectra of the complexes (**a**) Eu(TTA)_2_(phen)UA, excited at 365 nm, and (**b**) Tb(*p*-BBA)_3_UA, excited at 280 nm.

The resolution was (1 nm, 1 nm), the monitoring wavelength was 613 nm and the excitation spectrum of the complex Eu(TTA)_2_(phen)UA was measured at room temperature, as shown in Figure 5a. The complex exhibits a broad excitation band of 200~400 nm with 365 nm as the optimal excitation peak. This is due to the fact that the ligands TTA and phen form a large conjugated structure after coordination with the Eu(III) ions, leading to a broad excitation band. The emission spectrum was measured with 365 nm as the excitation wavelength, as presented in Figure 5a. As can be seen from the figure, the characteristic emissions of Eu(III) ions are detected at 580, 591, 613 and 654 nm, corresponding to ^5^D_0_–^7^F_0_, ^5^D_0_–^7^F_1_, ^5^D_0_–^7^F_2_ and ^5^D_0_–^7^F_3_ level transitions of the Eu(III) ions, respectively. The ^5^D_0_–^7^F_2_ transition at 613 nm is the strongest, and the full width at half maximum (FWHM) is less than 5 nm, displaying high color purity. It is due to the fact that the ^5^D_0_–^7^F_2_ transition is a so-called “hypersensitive transition”, which means that its intensity is much more influenced by the local symmetry of the Eu(III) ions and the nature of the ligands than the intensities of the other electric dipole transitions. The ^5^D_0_–^7^F_1_ transition at 591 nm is a magnetic dipole-allowed transition, whose intensity is largely independent of the environment of the Eu(III) ions. The emissions at 580 nm and 654 nm are contributed to by the ^5^D_0_–^7^F_0_ and ^5^D_0_–^7^F_3_ electric dipole transitions, which are forbidden transitions according to the standard Judd–Ofelt theory, and it is assumed that the two transitions are due to J-mixing [26]. The luminescence process of the complex Eu(TTA)_2_(phen)UA can be explained in terms of energy transfer; the ligands TTA and phen absorb energy, electrons are excited from the ground state to the lowest singlet state, the energy is transferred to the lowest triplet state of the ligand by inter-system leap, then, the energy is transferred from the lowest triplet state of the ligands to the resonance energy level ^5^D_0_ of the Eu(III) ions by electron exchange and the 4f electrons of the Eu(III) ions emit characteristic light when they migrate back to the ground state by radiation.

The resolution was (1 nm, 1 nm) and the excitation spectrum of the complex Tb(p-BBA)_3_UA was measured and monitored at 545 nm, as illustrated in Figure 5b. The complex exhibits strong excitation bands between 200 and 400 nm with 280 nm as the optimal excitation peak. It is because the three ligands of p-BBA also form a large conjugated structure after coordination with the Tb(III) ions, producing a broad excitation band. The emission spectrum was excited by the wavelength of 280 nm, as shown in Figure 5b. The complex exhibits obvious characteristic emissions of the Tb(III) ions at 490, 545, 585 and 623 nm, which are attributed to f-f transition of the Tb(III) ions at ^5^D_4_–^7^F_6_, ^5^D_4_–^7^F_5_, ^5^D_4_–^7^F_4_ and ^5^D_4_–^7^F_3_. The strongest emission peak at 545 nm belongs to the hypersensitive transition [27], which shows strong sharp-line narrow-band pure green emission. The luminescence process of the complex Tb(p-BBA)_3_UA is similar to that of the complex Eu(TTA)_2_(phen)UA, meeting the energy transfer requirement and thus exhibiting the characteristic emission of the Tb(III) ions.

### 3.5. Effect of Relative Molecular Weight on the Luminescent Properties of the Copolymer PMEuTb

The molecular weight and molecular weight distribution of the copolymer PMEuTb with different initiator concentrations were measured by a Waters 410 gel permeation chromatographer with tetrahydrofuran as an eluent, as shown in Table 2. With the increase of initiator concentration, the polymerization degree of the copolymer PMEuTb decreases and the number average molecular weight *M*_n_ and the weight average molecular weight *M*_w_ decrease. In the free radical polymerization, the higher the initiator concentration is, the more primary free radicals generate in the chain initiation stage and the more monomer free radicals form. However, the amount of the monomer is constant and the number of monomers on the molecular chain decrease, resulting in a decrease in molecular weight. On the other hand, the polymerization degree of the polymer in free radical polymerization is relevant to four facts: normal polymerization, transfer to the monomer, transfer to the initiator and transfer to the solvent. The monomer is methyl methacrylate, and its chain transfer constant to the monomer is small, about 10^−4^~10^−5^, which can be ignored. The initiator used in the experiment is AIBN. In this work, the chain transfer constant is 0.02, which is relatively small. The solvent in the experiment is dimethyl sulfoxide, there are no active hydrogen atoms or halogen atoms in the molecule and the chain transfer constant is relatively small. No chain transfer agent was added during the polymerization. Therefore, in this free radical polymerization system, the degree of polymerization of the polymer is only relevant to normal polymerization, and the chain termination is dominated by diradical termination. The higher the concentration of free radicals, the higher the probability of double group termination, and the chain termination stage comes earlier, resulting in the decrease of molecular weight. The molecular weight distribution index of the copolymer is 1.5~2.0, demonstrating that the molecular weight distribution is narrow and the molecular weight distribution is relatively uniform.

**Figure 6:**
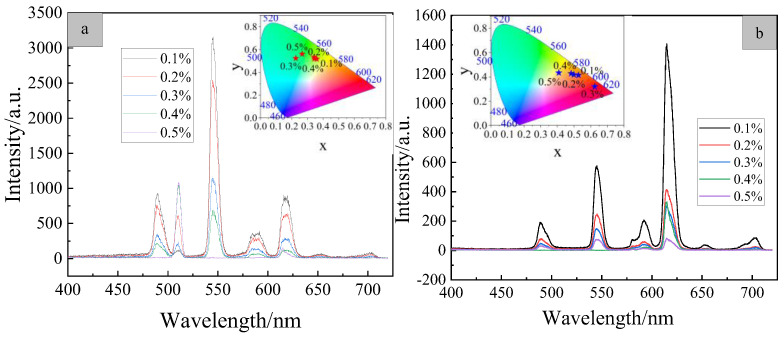
The luminescent spectra of serial copolymer PMEuTb excited by 254 nm (**a**) and 365 nm (**b**).

In order to investigate the effect of different initiator concentrations on the luminescent properties, the excitation wavelength was set at 254 nm, the resolution was set at (5.0, 5.0 nm) and the phosphorescent emission spectra of serial copolymers PMEuTb with different initiator concentrations were recorded at room temperature in solid state, as presented in Figure 6a. It can be learned that the serial copolymers exhibit characteristic emission at 489, 545, 589, 615 and 650 nm. The emission peaks at 489 and 545 nm are attributed to the characteristic emission of the Tb(III) ions, corresponding to the ^5^D_4_–^7^F_6_ and ^5^D_4_–^7^F_5_ transitions of the Tb(III) ions; the emission peak at 650 nm is attributed to the characteristic emission of the Eu(III) ions, corresponding to the ^5^D_0_–^7^F_3_ transition of the Eu(III) ions. The emission peak at 589 nm has a wider FWHM, which is transformed from sharp line emission in the complex to broad peak emission, in that this peak is the superposition of emission peaks at 591 nm (^5^D_0_–^7^F_1_ transition) in the Eu(III) ions and at 585 nm (^5^D_4_–^7^F_4_ transition) in the Tb(III) ions. FWHM at 615 nm also significantly increases, which is the result of the superposition of the emission peaks at 613 nm (^5^D_0_–^7^F_2_ transition) in the Eu(III) ions and at 623 nm (^5^D_4_–^7^F_3_ transition) in the Tb(III) ions. The sharp line emission at 510 nm does not appear in the complexes Eu(TTA)_2_(phen)UA and Tb(*p*-BBA)_3_UA, and it is the frequency-doubling emission at the excitation wavelength of 254 nm.

Comparing the luminescent intensity of the serial copolymers PMEuTb with different initiator concentrations, the general trend is that luminescent intensity decreases with the increase of the initiator concentration. Combined with the molecular weight and distribution of different initiator concentrations, the relative molecular weight of the polymer decreases and the molecular weight distribution gradually increases with the increase of the initiator concentration, indicating that, with the increase of the initiator concentration, the molecular chain gradually is shortened, resulting in more and more dense distributions of complexes in the polymer molecular chain and a non-radiation transition probability increase. Therefore, the luminescent intensity of the serial copolymer PMEuTb decreases with the increase of the initiator concentration. The luminescent intensity of the copolymer with an initiator concentration of 0.1% is the highest. In the subsequent experiments, the copolymer with an initiator concentration of 0.1% is selected for further research.

The phosphorescent emission spectra of the serial copolymer PMEuTb with different initiator concentrations were measured by 365 nm of ultraviolet light at the resolution (5.0, 5.0 nm), as shown in Figure 6b. The serial copolymers exhibit characteristic emission at 489, 545, 589, 615 and 650 nm. The emission peaks at 489 and 545 nm correspond to the ^5^D_4_–^7^F_6_ and ^5^D_4_–^7^F_5_ transitions of the Tb(III) ions and the emission peak at 650 nm is attributed to the ^5^D_0_–^7^F_3_ transitions of the Eu(III) ions. The FWHM at 589 nm is wider, which is the superposition of emission peaks of the ^5^D_0_–^7^F_1_ transition of the Eu(III) ions at 591 nm and the ^5^D_4_–^7^F_4_ transition of the Tb(III) ions at 585 nm. Different from the emission spectra excited at 254 nm, the maximum emission intensity is located at 615 nm, being pure red-light emission.

According to the phosphorescent emission spectra of the series of copolymers, their CIE color coordinates were calculated, as shown in respective subgraphs of Figure 6. When the excitation wavelength is 254 nm, its color coordinate is located in the green region because the emission intensity of the green complex Tb(*p*-BBA)_3_UA is 3597, while that of the red complex Eu(TTA)_2_(Phen)UA’s emission intensity is only 788. When the excitation wavelength is set as 365 nm, its color coordinate is in the red-light region. Therefore, when 365 nm is used as the excitation wavelength, its luminescence displays mainly red-light emission. These data enable us to conclude that the copolymer PMEuTb has dual-wavelength emission characteristics.

### 3.6. Luminescent Properties of the Copolymer PMEuTb with Initiator Concentration of 0.1%

**Figure 7:**
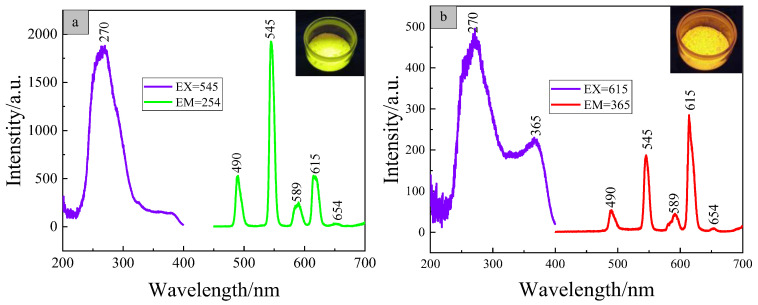
The luminescent spectra and luminescent photos of copolymer PMEuTb with initiator concentration of 0.1% under excitation wavelengths 254 nm (**a**) and 365 nm (**b**).

The copolymer PMEuTb with an initiator concentration of 0.1% was selected as the research object, the resolution was set as (1.0 nm, 1.0 nm) and the excitation spectra were measured with 615 nm and 545 nm as the monitoring wavelengths, as shown in Figure 7. The copolymer PMEuTb exhibits a strong excitation band at 220~400 nm, mainly due to the joint action from the complexes Tb(*p*-BBA)_3_UA and Eu(TTA)_2_(phen)UA. The optimal excitation wavelengths of the copolymer PMEuTb are both located at 270 nm under the monitoring of 615 nm and 545 nm wavelengths. Compared with the monomers, the optimal excitation wavelengths are all blue-shifted. In addition, the excitation wavelengths change from 285 nm and 365 nm in the complex Eu(TTA)_2_(phen)UA to 270 nm and 365 nm in PMEuTb, and the optimal excitation wavelength changes from 365 nm to 270 nm. It may be because the ligand *p*-BBA of Tb(III) ions indirectly excited the Eu(III) ions after the red and green light complex monomers were copolymerized with methyl methacrylate, resulting in the excitation wavelength scope and optimal excitation peak at the monitoring wavelength of 615 nm being consistent with that of 545 nm.

Since 254 nm and 365 nm UV lamps are common on the market, the emission spectrum of the copolymer was measured by using excitation wavelengths of 254 nm and 365 nm, as shown in Figure 7. As can be seen from the figure, both spectra show characteristic emission peaks at 490, 545, 589, 615 and 654 nm. Among them, the emission peaks at 490 nm and 545 nm are attributed to the characteristic emission of the Tb(III) ions. The peaks at 615 nm and 654 nm correspond to the characteristic emission of the Eu(III) ions. The emission peak at 589 nm has a wider FWHM, which is the result of superposition at the 591 nm of the Eu(III) ions and the 585 nm of the Tb(III) ions. Compared with the complex Eu(TTA)_2_(phen)UA, characteristic emission at 613 nm redshifts to 615 nm in the copolymer PMEuTb, still maintaining the characteristic sharp line emission. This is owing to the fact that the *f*-*f* level transition of ^5^D_0_–^7^F_2_ is a hypersensitive transition and is easily affected by surroundings, leading to a slight red shift of characteristic emission in the copolymer. The difference is that the luminescent performance of the emission spectrum is different with different excitation wavelengths. Under 254 nm excitation, the green-light emission peak of 545 nm is strong and the red-light emission peak of 615 nm is weak, demonstrating green-light emission in whole. However, under 365 nm excitation, the red-light emission peak of 615 nm is strong and the green-light emission peak is relatively weak, displaying red-light emission in whole, as shown in the color of the luminescent photo in Figure 7. Therefore, the copolymer is a kind of dual-wavelength bonded luminescent material, which can be used to prepare dual-wavelength anti-counterfeit fibers.

### 3.7. Variation-Temperature Luminesecent Spectra of the Copolymer PMEuTb

**Figure 8:**
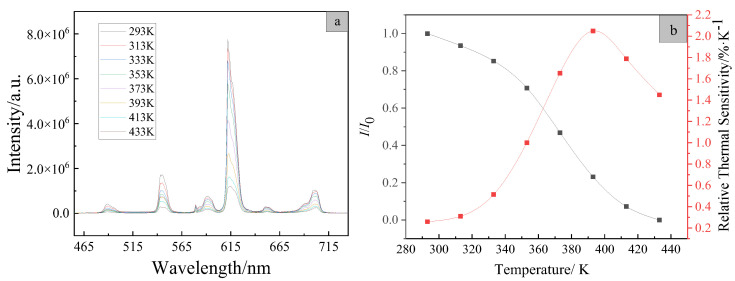
The variable-temperature luminescent spectra (**a**); the plots of luminescent intensity and relative thermal sensitivity as a function of temperature at 615 nm of the copolymer PMEuTb (**b**).

A variable temperature luminescent spectrum is one of the key indicators of thermal quenching performance of luminescent materials. In order to investigate the thermal quenching properties of the copolymer PMEuTb, taking 365 nm as an example, its temperature-variable luminescent spectra were measured in the range of 293~433 K, as shown in Figure 8a. The copolymer mainly exhibits red-light emission of the Eu(III) ions under the excitation of 365 nm, which is the optimized excitation wavelength of the complex Eu(TTA)_2_(phen)UA, resulting that the luminescent intensity in the copolymer at 615 nm is the strongest. The luminescent intensity at 615 nm is the research object, and the plot of the variation of integrated area at 615 nm with temperature is shown in Figure 8b.

The relative thermal sensitivity (*S*_r_) is calculated by the following Equation (1) [28]

(1) $$S_{r}=\frac{\left(\frac{\partial \Delta }{\partial T}\right)}{\Delta }$$

where ∆ is the thermometric parameter (in this case, the integrated area of the emission peak at 615 nm) at a certain temperature and *∂*∆ is the variation of this signal upon a certain temperature variation (*∂T*). The plot of the dependence of relative thermal sensitivity *S*_r_ on *T* is presented in Figure 8b. The figure shows that the emission intensity at 615 nm gradually decreases with the increase of temperature. At 313 K, the luminescent intensity decreases very little and remains at 94% of the initial luminescent intensity and the relative thermal sensitivity *S*_r_ is only 0.31%•K^−1^. At 333 K, the luminescent intensity decreases slowly and the copolymer PMEuTb remains at 85% of the initial luminescent intensity with 0.51%•K^−1^ of *S*_r_. The results suggest that the luminescent properties of the copolymer are not affected much by the temperature before 333 K, meet the requirements of room temperature phosphorescent anti-counterfeiting materials [29]. Starting at 373 K, luminescent intensity acceleratingly declines, which results in only 25% of the initial luminescent intensity at 433 K. The reason for this result is that the copolymer PMEuTb is an amorphous polymer, and the glass transition temperature *T*_g_ of the polymethyl methacrylate is around 378 K [30]. When the temperature is lower than *T*_g_, the polymer is in a glass state, the molecular chains and segments keep the static and the monomer units of the red-light and green-light complexes are well fixed in the specific position of the polymer molecular chain. However, the vibration amplitude of atoms (or groups) in the equilibrium position of molecules is aggravated with the increase of temperature, which leads to the decrease of luminescent intensity, but the decrease is not very obvious. However, when the temperature rises above *T*_g_, the molecular chain segments begin to move, and the polymer is in a highly elastic state. Due to the increased movement of the chain segment, the “cage” effect of the polymer chain segment on the luminescent group is weakened, and the collision probability and non-radiative transition of the luminescent group increase, leading to a sharp decline in luminescent intensity. With the further increase of temperature, the polymer enters the viscous state, the whole molecular chain starts to stretch and move and the luminescent performance of the copolymer is reduced by the influence of the temperature, which displays the decrease of relative thermal sensitivity.

### 3.8. Luminescent Lifetime of the Copolymer PMEuTb

Using an Edinburgh Instruments FLS980 steady-state/transient fluorescent spectrometer, time-dependent single photon counting (TCSPC) was used to monitor the phosphorescent decay process of the Eu(III) and Tb(III) ions in the luminescent centers of the complex and copolymer. According to Equation (2), double exponential fitting was performed. Then, the average phosphorescent lifetime *τ* of the phosphorescent decay curve was calculated according to Equation (3) [31].

(2) $$I\left(t\right)=A+B_{1}\exp\left(-t/\tau_{1}\right)+B_{2}\exp\left(-t/\tau_{2}\right)$$

(3) $$\tau =\frac{B_{1}\tau_{1}^{2}+B_{2}\tau_{2}^{2}}{B_{1}\tau_{1}+B_{2}\tau_{2}}$$

where *τ*_1_ and *τ*_2_ are short and long lifetime, respectively, and *B*_1_ and *B*_2_ are fitting constants.

**Figure 9:**
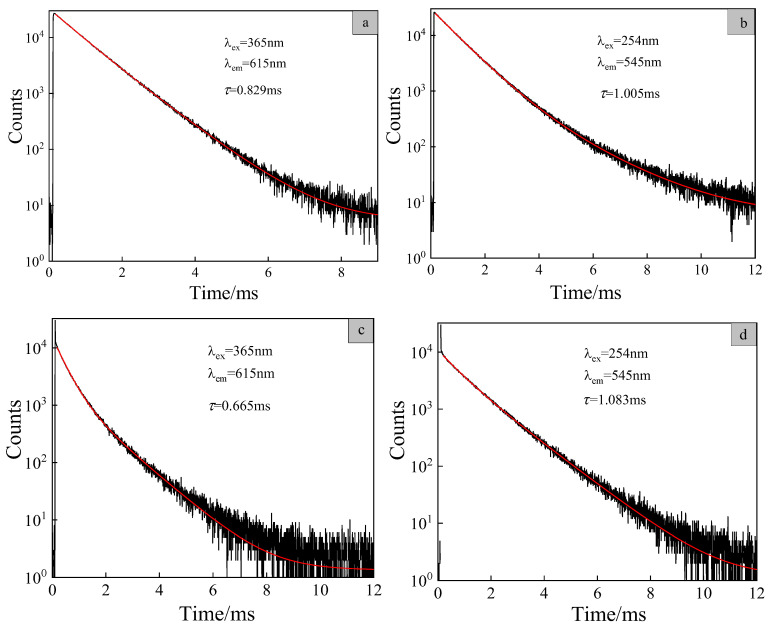
Phosphorescent decay curves of the ^5^D_0_–^7^F_2_ transition of the Eu(III) ions in Eu(TTA)_2_(phen)UA (**a**), the ^5^D_4_–^7^F_5_ transition of the Tb(III) ions in Tb(*p*-BBA)_3_UA (**b**), the ^5^D_0_–^7^F_2_ transition of the Eu(III) ions (**c**) and the ^5^D_4_–^7^F_5_ transition of the Tb(III) ions (**d**) in PMEuTb.

The excitation wavelength was set as 365 nm, and the phosphorescent decay process of the ^5^D_0_–^7^F_2_ transition of Eu^3+^ in the complex Eu(TTA)_2_(phen)UA and the copolymer PMEuTb was monitored, as shown in Figure 9a,c. The fitting analysis shows that, in the complex, *A* = 5.306, *B*_1_ = 18536.23, *B*_2_ = 7688.68, *τ*_1_ = 0.71499 ms, *τ*_2_ = 1.02338 ms and the average lifetime of the complex Eu(TTA)_2_(phen)UA calculated by Equation (3) is 0.829 ms. As for the copolymer, *A* = 1.346, *B*_1_ = 8336.19, *B*_2_ = 1943.81, *τ*_1_ = 0.38735 ms, *τ*_2_ = 1.08867 ms and the average fluorescence lifetime of the ^5^D_0_–^7^F_2_ transition of the Eu(III) ions in copolymer PMEuTb was calculated by Equation (3) to be 0.665 ms (the lifetime is between 10^−6^ and20 s, which is attributed to the phosphorescent materials). Compared with the complex Eu(TTA)_2_(phen)UA, the phosphorescent lifetime decreases from 0.829 ms to 0.665 ms in the copolymer, indicating that the molecular structure of the copolymer has changed. The Eu(III) ions are sensitive to environmental disturbance, and the change of microenvironment leads to the increase of the phosphorescent emission rate constant, so the phosphorescent lifetime of the Eu(III) ions decreases.

The excitation wavelength was set as 254 nm, and the phosphorescent decay process of the ^5^D_4_–^7^F_5_ transition of the Tb(III) ions in the complex Tb(*p*-BBA)_3_UA and the copolymer PMEuTb was monitored, as shown in Figure 9b,d. The fitting analysis shows that, in the complex, *A* = 5.878, *B*_1_ = 23109.10, *B*_2_ = 1799.65, *τ*_1_ = 0.8548 ms, *τ*_2_ = 1.8801 ms and the average lifetime of the complex Tb(*p*-BBA)_3_UA calculated by Equation (3) is 1.005 ms. For the copolymer, *A* = 1.164, *B*_1_ = 3682.83, *B*_2_ = 5084.70, *τ*_1_ = 0.6495 ms, *τ*_2_ = 1.2463 ms and the average phosphorescent lifetime of the ^5^D_4_–^7^F_5_ transition of the Tb(III) ions in the copolymer PMEuTb was calculated by Equation (3) to be 1.083 ms. Compared with the complex Tb(*p*-BBA)_3_UA, the phosphorescent lifetime of the Tb(III) ions in the copolymer PMEuTb increases, while the phosphorescent lifetime of the Eu(III) ions decreases, which may be caused by the energy transfer from the Eu(III) ions to the Tb(III) ions [32].

### 3.9. Anti-Counterfeiting Application of the Copolymer PMEuTb

**Figure 10:**
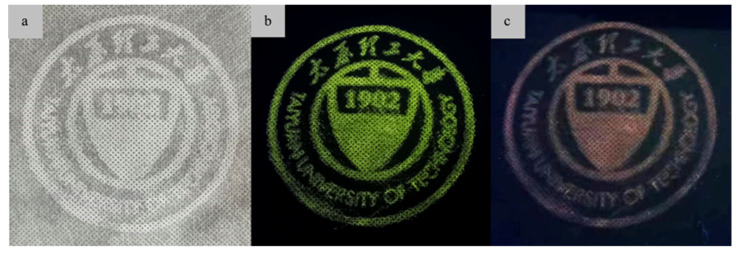
Photo of non-woven fabric printed with PMEuTb phosphors under natural light (**a**) and UV light at 254 nm (**b**) and 365 nm (**c**).

In this work, the copolymer phosphor PMEuTb has the advantages of good solubility, film-forming performance and photochromic properties, good compatibility with textiles and paper and has potential application value in the field of anti-counterfeiting, luminous fabric and intelligent textile applications. Employing standard screen-printing techniques, the luminescent ink prepared by PMEuTb phosphors was added to the screen printing plate containing the logo and then squeezed with an extruder to squeeze the luminescent ink onto the non-woven fabric below the printing plate. The optical photo of the prepared screen-printed logo under visible light is shown in Figure 10a. Under natural light, the looming logo pattern can be seen on the non-woven fabric, which has a certain degree of concealment. The logo pattern was irradiated with 254 nm and 365 nm UV lamps in a dark environment. The actual photo is shown in Figure 10b,c. The school badge pattern appears green under 254 nm light and cuticolor under 365 nm light. In addition, the green and cuticolor logos have better shape definition and clearly show the details of the anti-counterfeiting mark, indicating that the prepared PMEuTb phosphors have potential application value in phosphorescent anti-counterfeiting applications.

## 4. Conclusions

The present work has developed a phosphorescent anti-counterfeiting copolymer PMEuTb with dual-wavelength excitation and dual-wavelength emission employing the technique of first coordination and then polymerization to overcome the problem of single-wavelength excitation of polymerization and then coordination, which improves the anti-counterfeiting level. The bonded molecular structure of the copolymer phosphor PMEuTb was confirmed by the infrared and ultraviolet spectra. The number average molecular weight *M*_n_ of the copolymer decreases with the increase of the initiator concentration. The luminescent properties of the copolymer are positively correlated with *M*_n_. The copolymer PMEuTb exhibits two colors of green light and red light under the excitation of ultraviolet light at 254 nm and 365 nm, and its temperature-variable luminescent spectra indicate that the luminescent intensity remains at 85% of the original at 333 K, which meets the requirements of room temperature phosphorescent anti-counterfeiting materials. The luminescent pattern fabricated by standard screen printing shows clearly visible green and cuticolor logos excited by 254 nm and 365 nm, indicating that the bonded copolymer phosphors have potential applications in phosphorescent anti-counterfeiting. In order to further improve the level of anti-counterfeiting, research on multi-wavelength phosphorescent anti-counterfeiting materials for red, green and blue will be carried out in the follow-up work.

## Figures and Tables

**Table 1:** The characteristic peaks of Eu(TTA)_2_(phen)UA, Tb(*p*-BBA)_3_UA and ligands.

Assignment (cm^−1^)	TTA *	phen *	*p*-BBA *	UAH *	Eu(TTA)_2_(phen)UA	Tb(*p*-BBA)_3_UA
ν_C=O(keto_-_)_	1588		1652		1600	1591
ν_C=O(COOH)_			1680	1710		
ν_C_-_O(COOH)_			1289	1268		
ν_as(COO_-_)_					1535	1543
ν_s(COO_-_)_					1413	1417
ν_C=N_		1485			1462	
ν_CF3_					1242, 1190, 1138	
ν_Eu_-_N_					460	
ν_Eu_-_O_					493	
ν_Tb_-_O_						495
ν_C=C(UA)_						1654

* Infrared spectra data are freely accessed from http://www.chemcpd.csdb.cn/, accessed on 1 August 2022.

**Table 2:** Molecular weight and its distribution of PMEuTb with different initiator concentrations.

PMEuTb	*M*_n_/kg•kmol^−1^	*M*_w_/kg•kmol^−1^	*M*_w_/*M*_n_
1 (0.1%)	84,351	146,481	1.7
2 (0.2%)	57,973	110,424	1.9
3 (0.3%)	33,523	87,043	2.6
4 (0.4%)	16,243	39,461	2.4
5 (0.5%)	7654	23,431	3.0

## Data Availability

The data is unavailable due to privacy restrictions.
